# Estimation of exogenous drivers to predict COVID-19 pandemic using a method from nonlinear control theory

**DOI:** 10.1007/s11071-021-06811-7

**Published:** 2021-09-06

**Authors:** Christoph Hametner, Martin Kozek, Lukas Böhler, Alexander Wasserburger, Zhang Peng Du, Robert Kölbl, Michael Bergmann, Thomas Bachleitner-Hofmann, Stefan Jakubek

**Affiliations:** 1 Institute of Mechanics and Mechatronics, TU Wien, Getreidemarkt 9, 1060 Vienna, Austria; 2 Division of Visceral Surgery, Department of General Surgery, Medical University of Vienna, Vienna, Austria; 3 Comprehensive Cancer Center, Medical University of Vienna, Vienna, Austria

**Keywords:** SARS-CoV2, COVID-19, Epidemiological modelling, Differential flatness, Dynamical systems

## Abstract

The currently ongoing COVID-19 pandemic confronts governments and their health systems with great challenges for disease management. Epidemiological models play a crucial role, thereby assisting policymakers to predict the future course of infections and hospitalizations. One difficulty with current models is the existence of exogenous and unmeasurable variables and their significant effect on the infection dynamics. In this paper, we show how a method from nonlinear control theory can complement common compartmental epidemiological models. As a result, one can estimate and predict these exogenous variables requiring the reported infection cases as the only data source. The method allows to investigate how the estimates of exogenous variables are influenced by non-pharmaceutical interventions and how imminent epidemic waves could already be predicted at an early stage. In this way, the concept can serve as an “epidemometer” and guide the optimal timing of interventions. Analyses of the COVID-19 epidemic in various countries demonstrate the feasibility and potential of the proposed approach. The generic character of the method allows for straightforward extension to different epidemiological models.

## Introduction

Epidemics and their severe socio-economic impact on society have been the focus of scientific research for centuries [1–3]. The primary goal is to find new and better approaches to model and to reduce the spread of infectious diseases. In this context, this paper proposes a method from nonlinear control theory that can be applied to common compartmental models [4, 5] in order to estimate their epidemiological states and exogenous variables in real time. As a main contribution, it is demonstrated how this method can be used for the analysis and prediction of multiple epidemic surges.

In epidemiological modelling, the most widespread class is that of compartmental models [6], of which the best-known representative is the SIR model defined by Kermack and McKendrick in 1927 [7, 8]. Since then, a variety of compartmental models has been derived to account for numerous infectious diseases. The basic SIR model has often been adapted or expanded by researchers for different purposes. A well-known representative is the SEIR model [9, 10], which is often used as a basis for further extensions [11–13]. Overall, the modification of compartmental models has led to a great variety of available epidemiological models, the discussion of which would be beyond the scope of this paper [14–21].

An important aspect in epidemiological modelling is the differentiation between endogenous and exogenous variables. *Exogenous* variables can be seen as independent variables or outside drivers that often have a significant impact on *endogenous* variables. This distinction goes along with the notion of causality: Exogenous variables are thought of as causes, endogenous variables as their effects.

Although exogenous variables can significantly affect the dynamics of epidemiological models, they are often difficult or impossible to determine. This is particularly true for models of COVID-19 epidemics where the exogenous drivers can comprise factors such as mobility and changes in social behaviour [22–24]. In the actual COVID-19 pandemic, there are also additional factors, resulting from intervention measures such as the obligation to wear face masks, governmental restrictions on commerce or curfews [11, 25–33]. While it is generally known, *when* these measures were imposed, their *quantitative* impact can only be assessed with great difficulty or delays. For instance, specific social interactions in the ongoing COVID-19 pandemic can cause linear infection curves which cannot be modelled with conventional compartmental models [24].

This paper presents an approach to estimate an aggregated exogenous driver of an epidemic. It is based on a method from nonlinear control theory which can be combined with many existing compartmental models, specifically including SEIR or SIR with dead time [34]. It will be demonstrated that through this concept extensive and significant insights into the epidemics can already be obtained using the basic SIR model. Using the reported infection cases as the only data source, an estimate of the exogenous drivers can be obtained in real time, leading to more accurate and dependable prognoses. Our analysis shows that exogenous drivers are mainly responsible for the onset of epidemic waves such as COVID-19. While state-of-the-art models would not predict epidemic waves on their own, the proposed approach can already provide an early indication for an upcoming wave, especially when the number of infections is still inconspicuously low.

Based on a quantitative analysis, it is shown, that utilizing the proposed approach a time-varying transmission rate can be neglected in explaining the COVID-19 dynamics. This is even true for the impact of mutations, which is also shown in the results. In addition, it is shown how short-term scenarios of hypothetical non-pharmaceutical interventions based on compartmental models could provide a more comprehensive basis for governmental policy-making.

This paper is structured as follows: First, the fundamental model structure is introduced and the effects of exogenous variables for compartmental models are illustrated. Next, the exogenous drivers are derived, including an investigation of a time-variant transmission rate. Finally, the exogenous drivers are applied to the epidemics of various countries and the results are discussed.

## Compartmental models with exogenous inputs

Compartmental models are widely used to analyse, simulate and predict epidemic waves [4]. In particular, they are extensively used to predict the spread of COVID-19 in several countries, [35, 36]. This section describes the extension of compartmental models to enable the estimation of exogenous drivers. Subsequently, the concept of differential flatness is used to determine the current state and the exogenous input directly from the measured infection numbers.

### Exogenous input and contact-less compartment

With only a few exceptions (e.g. [22, 37, 38]), compartmental models constitute autonomous differential equations with no exogenous inputs. Therefore, their predictions mainly depend on the chosen initial conditions. However, nearly all dynamic systems are driven by *exogenous* inputs that are either not measurable or unknown.

**Fig. 1:**
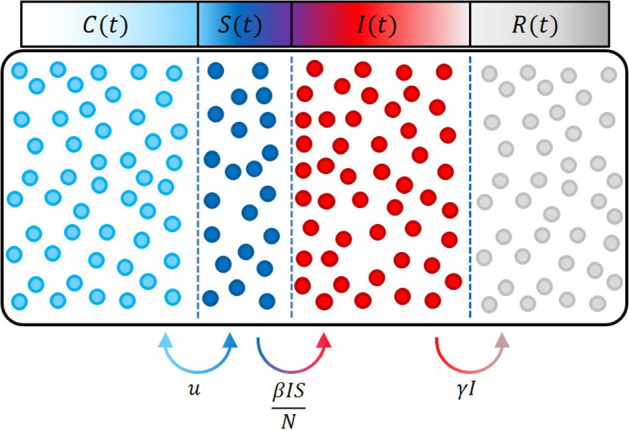
CSIR compartment model with flows of individuals between the compartments. While the transmission of individuals from *S* to *I* and from *I* to *R* is unidirectional, the aggregated exogenous drivers *u* can act bidirectionally on the system

The investigations presented in this paper are based on input-affine compartmental models of the form:

1 $$\begin{aligned} \begin{aligned} {\dot{\mathbf {x}}}&= {\varvec{f}}({\mathbf {x}}) + {\varvec{g}}({\mathbf {x}}) u \\ y&= h({\mathbf {x}}). \end{aligned} \end{aligned}$$

Here, ${\mathbf {x}}$ are the epidemiological states, *y* is recorded output (i.e. the case numbers), *u* is the exogenous input, ${\varvec{f}},{\varvec{g}}$ are vector fields and *h* is a nonlinear function. Autonomous systems therefore are missing the expression ${\varvec{g}}({\mathbf {x}}) u$. The general representation of system (1) applies in particular to, e.g. the standard SIR or SEIR compartmental models. For the sake of clarity, in the following the proposed methods are demonstrated by means of the SIR model for which the corresponding formulation is:

2 $$\begin{aligned} \left\{ \begin{array}{l} {\dot{S}} = - \frac{\beta I S}{N} + u(t) \\ {\dot{I}} = \frac{\beta I S}{N} -\gamma I \\ {\dot{R}} = \gamma I \end{array}\right. . \end{aligned}$$

In (2), *S*(*t*) is the number of susceptible individuals, *I*(*t*) is the number of currently infected individuals, and *R*(*t*) is the number of recovered individuals, respectively, at time *t*. The additional input *u*(*t*) represents aggregated exogenous drivers can be interpreted as the flow of individuals per time period moving into or out of compartment *S*, see Fig. 1.

For instance, the effect of social distancing can thus be captured via this input. In this case, individuals are moved into compartment *C* (in Fig. 1) and classified as *contact-less* individuals.

Although system (2) looks similar to the classical SIR model, the population *N* is no longer required to be equal to the sum of the compartments *S*, *I*, *R*, thus

3 $$\begin{aligned} N \ne S+I+R. \end{aligned}$$

Rather, with *C* constituting an additional compartment, the equality constraint can be reformulated as

4 $$\begin{aligned} N = C+S+I+R. \end{aligned}$$

In this CSIR model, no additional state equation for *C* is necessary as $C=N-S-I-R$ is uniquely defined. However, *S* is no longer strictly monotonically decreasing but contains essential information on the dynamics, and the unknown initial condition $S_0$ of *S* has to be estimated from data.

The extension of compartmental models with *C* and augmenting an exogenous driver *u*(*t*) is not limited to the standard SIR model, since all compartmental models feature a compartment *S*. These adaptions are also possible for SEIR or other models with multiple additional compartments as shown in [39] for example.

### Example: multiple waves and state trajectories

The following example demonstrates how multiple pandemic waves can be explained by the exogenous driver *u*(*t*).

**Fig. 2:**
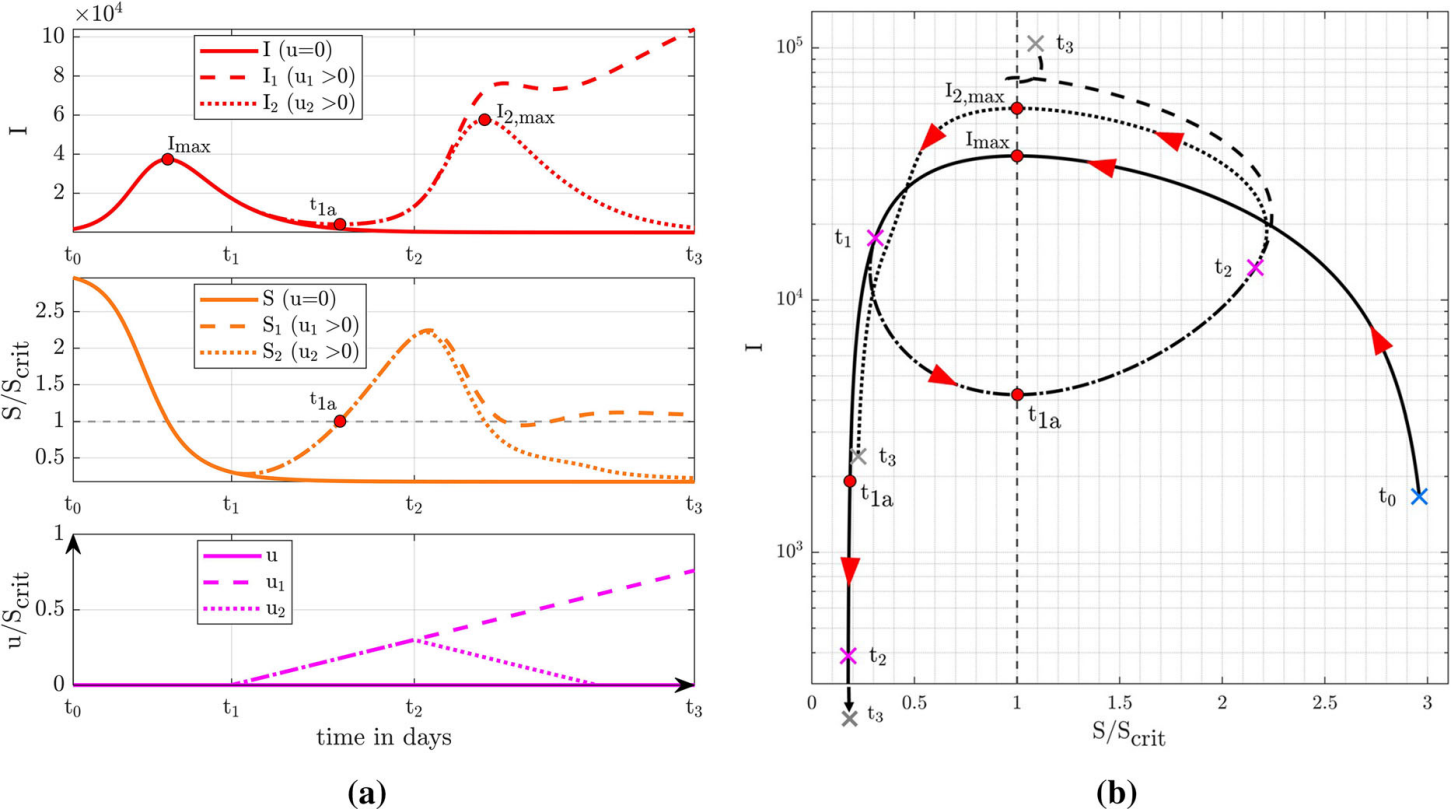
CSIR model with exogenous drivers describing the onsets of multiple epidemic waves (**a**) for different scenarios. Representation of the related infected and critical number of susceptibles for an epidemic in the state plane (**b**)

The effect of the exogenous input *u*(*t*) on the essential compartments *S* and *I* is illustrated in Fig. 2a. An epidemic wave, meaning exponential growth of *I*, occurs when a certain threshold in *S*(*t*) is exceeded [4, 40]. The critical value $S_{\text {crit}}$ is

5 $$\begin{aligned} S_{\text {crit}} = \frac{\gamma N}{\beta }. \end{aligned}$$

In the remainder $S_{\text {crit}}$ is utilized to normalize the number of susceptibles *S* and the exogenous drivers *u*(*t*). Fig. 2b shows the related state or phase diagram with the number of infections *I* over the relative susceptibility $S/S_{\text {crit}}$. As long as $u(t)=0$ holds, the epidemic follows exactly the course predicted by the original autonomous SIR model, leading to a maximum number of infections and a subsequent decrease as shown by the solid curves in *I*(*t*) and $S/S_{\text {crit}}$. In the state diagram in Fig. 2b, this is shown by the solid black trajectory.

For $u>0$ starting at $t_1$, the CSIR model (2) describes the possible onset of a new epidemic wave. Assuming a scenario where at time $t_1$, $u_1(t)$ and $u_2(t)$ start to grow linearly (dash-dotted line), the relative susceptibility starts to increase again despite still decreasing numbers of infections. At $t_{1a}$, the relative susceptibility reaches the critical value of one, which is followed by another surge in the numbers of infected individuals and—with a certain time lag—a second epidemic wave (shortly before $t_2$). The phase diagram in Fig. 2b depicts the scenario more clearly where the epidemic development leaves the original trajectory at $t_1$ in connection with the growing susceptibility. Once the susceptibility has reached the critical level of $S/S_{\text {crit}}=1$, another epidemic wave starts to develop.

More importantly, the origin of the second wave is triggered by the growth of the exogenous input $u_1(t)$, which already starts at $t_1$ where the infections of the first wave are still decreasing. This shows that there is a distinct order of events with a respective time lag between the increase in *u*(*t*), $S/S_{\text {crit}}$ and finally *I*(*t*).

At $t_2$, two alternative scenarios for *u*(*t*) are exemplified: a continuing linear growth ($u_1(t)$, dashed) and a linear decline to zero ($u_2(t)$, dotted). The continuing linear growth leads to an excessive epidemic surge, as observed with COVID-19 in many countries in late 2020. The linear decline of *u*(*t*) causes the epidemic to ease off. Both effects only become visible in the infection numbers after considerable delay.

## Estimation of unknown exogenous drivers of an epidemic through nonlinear control theory

This section describes how the aggregated exogenous driver *u*(*t*) can be estimated by a methodology from nonlinear control theory. This method requires the compartmental models to be differentially flat, which is addressed in the following.

### Concept of differential flatness

Differential flatness is a structural property of a class of nonlinear dynamical systems [41, 42]. It means that all state variables and inputs can be expressed as algebraic functions of so-called flat outputs and a finite number of their derivatives.

Various epidemiological compartment models (e.g. SIR, SEIR) have the property of differential flatness, which means that their states can be reconstructed by the measured number of infected and its time derivatives, respectively. For differentially flat systems, a virtual input *u*(*t*) can be constructed based on a purely mathematical recipe (e.g. [42]).

For many compartmental models, the flat input *u*(*t*) has a distinct and interpretable meaning: In the context of the CSIR model, it can be interpreted as a flux of individuals increasing or decreasing the number of susceptible individuals (Fig. 1). This can be caused by different effects: Reduced susceptibility due to social/physical distancing or lockdowns, increased susceptibility following the lifting of a lockdown, higher mobility or travel activities, etc.

### Estimation of states and inputs using the flatness property

For a differentially flat system (1), the state vector ${\mathbf {x}}$ and the flat input *u*(*t*) can be determined using only the measured *y*(*t*) and its time derivatives [41]:

6 $$\begin{aligned} {\mathbf {x}}(t)&= {\mathbf {\Psi }}_{\mathbf {x}} \left( y(t),\ {\dot{y}}(t),\ {\ddot{y}}(t)\right) \end{aligned}$$

7 $$\begin{aligned} u(t)&= {\mathbf {\Psi }}_u \left( y(t),\ {\dot{y}}(t),\ {\ddot{y}}(t)\right) . \end{aligned}$$

For model (2), the measured output *y*(*t*) is the number of reported or detected infected individuals $I_{\mathrm{d}}(t)$

8 $$\begin{aligned} y(t) = I_{\mathrm{d}}(t) = \varphi (t)I(t), \end{aligned}$$

where *I*(*t*) is the number of actually infected individuals and $\varphi (t)$ is the time-varying detection rate. For the sake of brevity of the presentation it is assumed that $\varphi = 1$ = const. and hence $I_{\mathrm{d}}(t) = I(t)$. However, in the Appendix, it is shown how event strongly time-varying detection rates can be considered properly in the analysis.

For the model structure (2) with unknown exogenous driver *u*(*t*) and a time-dependent transmission rate $\beta (t)$, a constant recovery rate $\gamma$ and *I* as the available output, the missing state variable *S* can be obtained using

9 $$\begin{aligned} {\dot{I}} = \frac{\beta I S}{N} -\gamma I. \end{aligned}$$

The state variable *S* is then given by

10 $$\begin{aligned} S = N\frac{{\dot{I}}+\gamma I}{\beta I}, \end{aligned}$$

which is a function of *I* and ${\dot{I}}$ in accordance with (6). Taking the time derivative of *S* gives

11 $$\begin{aligned} {\dot{S}}&= N \frac{{\ddot{I}}+\gamma I}{\beta I} - N{\dot{I}}\frac{{\dot{I}} + \gamma I }{\beta I^2} - N {\dot{\beta }} \frac{{\dot{I}} + \gamma I}{\beta ^2 I}. \end{aligned}$$

By inserting ${\dot{S}}$ from (2) into (11), an expression for *u* is obtained:

12 $$\begin{aligned} u = \frac{N}{\beta } \left( \frac{{\ddot{I}}}{I} - \frac{{\dot{I}}^2}{I^2} \right) + {\dot{I}} + \gamma I - N \frac{{\dot{I}} +\gamma I}{\beta I} \frac{{\dot{\beta }}}{\beta }. \end{aligned}$$

From this expression, *u*(*t*) can be determined directly from the differentially flat output and its derivatives, in accordance with (7). The determination of the flat input and the unknown state variable *S* was straightforward for the SIR model structure. For more complex model structures, the reader is referred to the literature [39, 43].

Using (10), (12) can be expressed more compactly:

13 $$\begin{aligned} u + S\frac{{\dot{\beta }}}{\beta } = \frac{N}{\beta } \left( \frac{{\ddot{I}}}{I} - \frac{{\dot{I}}^2}{I^2} \right) + {\dot{I}} + \gamma I. \end{aligned}$$

The two terms on left-hand side of (13) can be readily used to explain the course of the epidemic, represented by *I* and its derivatives on the right-hand side. First, the aggregated exogenous input *u*(*t*) can be interpreted as the contribution of the social behaviour of the population in terms of an inflow or withdrawal from the susceptible compartment, as outlined in Fig. 1. Second, the term $S {\dot{\beta }}/\beta$ expresses the effect of the change in transmission capability of the disease.

If the active case numbers *I*(*t*) and their time derivatives as well as $\beta (t)$ are available, (13) can be used directly for the estimation of the aggregated exogenous input *u*(*t*). While it is straightforward to obtain *I*(*t*) and its time derivatives, access to the generally time-varying transmission rate $\beta (t)$ in real time is rather involved and subject of intensive research [44, 45]. However, analysis of the epidemic courses in various countries in combination with reported variations of $\beta$ (e.g. due to mutations [46, 47]) showed, that its influence is almost negligible when estimating *u*(*t*) from (13). This will be substantiated in Sect. 3.3.2 where the effect of the spread of the significantly more infectious mutation B.1.1.7 on the estimate of *u*(*t*) is analysed. For the subsequent analysis, it is assumed, that $\beta$ is time invariant, hence ${\dot{\beta }}$=0. Then, *u*(*t*) is given by

14 $$\begin{aligned} u = \frac{N}{\beta } \left( \frac{{\ddot{I}}}{I} - \frac{{\dot{I}}^2}{I^2} \right) + {\dot{I}} + \gamma I. \end{aligned}$$

**Fig. 3:**
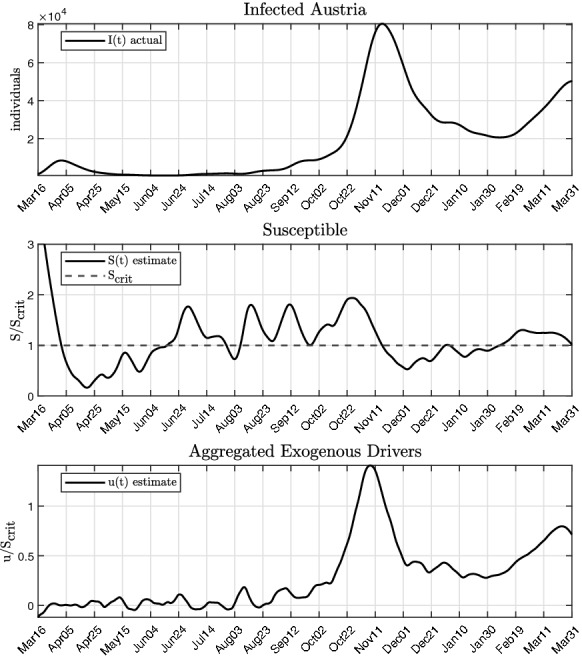
Estimated aggregated exogenous drivers *u*(*t*) for ${\dot{\beta }}$=0 and according compartment of susceptibles *S*(*t*) for Austria, both normalized by the critical value $S_\text {crit}$. The data of infected *I*(*t*) are based on the cases reported by the Austrian government [49]

In Fig. 3, the actual course of the active case numbers is shown in the top panel. Below, the flatness-based estimate of the susceptible compartment *S* from (10) is shown, whereby it was referenced to $S_{crit }$ from (5):

15 $$\begin{aligned} \frac{S}{S_crit } = \frac{{\dot{I}}}{\gamma I} +1. \end{aligned}$$

This allows an interpretation of $$S/S_crit $$ similar to the effective reproduction number $$R_eff $$ [48], as shown in Fig. 8. Finally, the bottom panel depicts the estimate of *u*(*t*), again referenced to $$S_crit $$. It can be seen that that most of the time $$u/S_crit $$ is positive, leading to a growth of the susceptible compartment, peaking at the end of October. This is followed by a phase of slowdown in December, where $$S/S_crit $$ is decreased significantly. In both cases, the exogenous input *u*(*t*) is ahead of the actual infection cases *I*(*t*) by approximately 2 weeks, thus serving as a leading indicator for the epidemic.

### Analysis of time-varying transmission rates

In this section, the effect of a time-varying transmission rate $\beta (t)$ is investigated and evaluated based on the course of the epidemics in Austria. The explanatory power of $\beta (t)$ is analysed and the effect of a mutation on $\beta (t)$ and subsequently on the estimate of *u*(*t*) is examined as well.

#### Using only $\beta (t)$ to explain the epidemic course

As the COVID-19 pandemic is now lasting for more than a year, in the literature various modelling approaches have been reported that involve repeated adaptation of the transmission rate $\beta$ [23, 50]. This is done to account for the highly dynamic epidemics and multiple epidemic waves in many countries around the globe. Fluctuations in the transmission rate are also often used to explain the effectiveness of non-pharmaceutical interventions such as lockdowns, [50]. In this case, the transmission rate is used to explain both the transmission capability of the virus and the social behaviour of the population, e.g. following a lockdown.

In this subsection, it is therefore demonstrated how the flatness-based approach could also be used to provide for a continuous real-time estimate of $\beta$. To this end, it now assumed that the epidemic course can be explained solely by a time-varying transmission rate $\beta (t)$, hence $u(t) \equiv 0$. Then, Eq. (13) becomes

16 $$\begin{aligned} S\frac{{\dot{\beta }}}{\beta } = \frac{N}{\beta } \left( \frac{{\ddot{I}}}{I} - \frac{{\dot{I}}^2}{I^2} \right) + {\dot{I}} + \gamma I. \end{aligned}$$

Inserting *S*(*t*) from (10) and rearranging for ${\dot{\beta }}$ yields

17 $$\begin{aligned} {\dot{\beta }} = \frac{\beta I}{{\dot{I}} +\gamma I} \left( \frac{{\ddot{I}}}{I} - \frac{{\dot{I}}^2}{I^2} \right) + \frac{\beta ^2 I}{N}. \end{aligned}$$

**Fig. 4:**
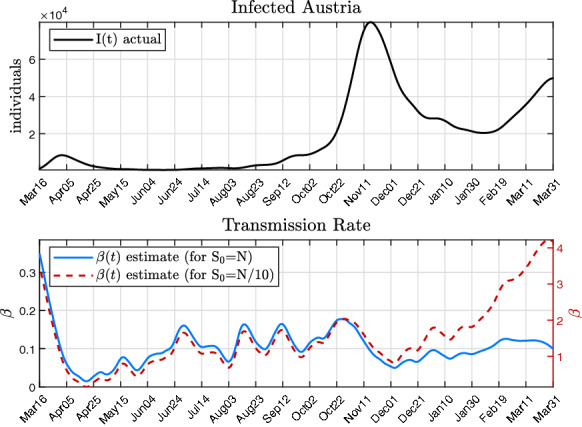
Results of multiple epidemic waves described by a time-varying transmission rate $\beta (t)$ with *u*(*t*)=0 and for different initial conditions $S_0$. The data of infected *I*(*t*) are based on the cases reported by the Austrian government [49]

With the actual course of the epidemic, given by *I*(*t*) and its derivatives, Eq. (17) constitutes a first order differential equation for $\beta (t)$. The initial condition $\beta _0$ is determined from Eq. (10), after an initial size $S_0$ of the susceptible population has been chosen. SIR models generally assume that the entire population is susceptible at the beginning of an epidemic, i.e. $S_0$=*N* [4]. A corresponding course of $\beta (t)$ from Eq. (17) using actual epidemic data from Austria is shown in Fig. 4 (blue) in the second panel. For comparison, an alternative solution of $\beta (t)$ is shown, assuming that only a fraction of the population is in the susceptible compartment at the beginning of the epidemic (i.e. $S_0$=*N*/10).

The estimates of $\beta (t)$ which are obtained from (17) confirm that a time-varying transmission rate is in fact able to reproduce the dynamics of *I*(*t*). The results suggest that the transmission rate has to fluctuate continuously and in a wide range in order to explain *I*(*t*).

#### Influence of mutations on the estimate of *u*(*t*)

In the previous section, it was stated that the variability of the transmission rate has only little effect on the flatness-based estimate of the exogenous input. Hence, in Fig. 3, a first analysis was given which was based on the assumption that ${\dot{\beta }}=0$.

It is now analysed how this analysis would have been affected by the rapid onset and spread of a new virus strain which is 70% more infectious than its predecessor, e.g. the British mutation B.1.1.7 [46]. It is further assumed that it becomes the dominant variant after some time *T*=90 days. To this end, the transmission rate $\beta _\text {m}(t)$ is represented by as

18 $$\begin{aligned} \beta _\text {m}(t) = \beta _0 + \varDelta \beta \frac{1}{1 + e^{-(t-t_0)/T}}, \end{aligned}$$

where $\beta _0$ is the initial transmission rate, $\varDelta \beta$ is the net increase in the transmission rate of the new strain, *T* is the time for the new mutation to replace its predecessor and $t_0$ marks the time when the initial virus has been replaced by 50%.

Replacing $\beta (t)$ in (13) by (18) results in a new estimate for *u*(*t*) that accounts for the rapid spread of the new mutation.

**Fig. 5:**
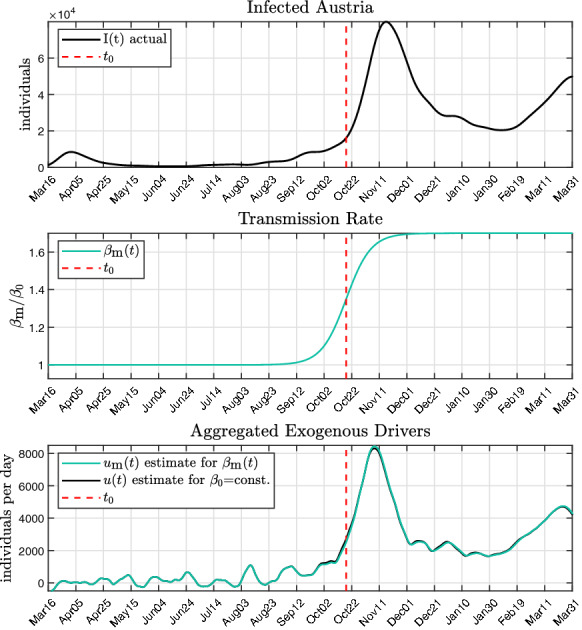
Transmission rate and exogenous drivers $u_\text {m}(t)$ for the mutation. The exogenous drivers $u_\text {m}(t)$ are compared to *u*(*t*) of Fig. 3 with $\beta _0$=const. The data of infected *I*(*t*) are based on the cases reported by the Austrian government [49]

The effects of such a mutation for the estimation of $u_\text {m}(t)$ are shown in Fig. 5. The estimate of the exogenous driver *u*(*t*) is compared to that which was obtained from (14) where $\beta (t)$=$\beta _0$=const. was assumed. Obviously, the impact of the mutation on the estimate of the exogenous driver is minimal, although the assumed mutation scenario is significant, resulting in a 70% surge of the transmission number in only 90 days. Based on these results, it is reasonable to assume that the estimate of the exogenous input *u*(*t*) is sufficiently accurate using Eq. (14) where a constant transmission rate ${\beta }$ is assumed for each country.

## Analysis of several countries

### Prediction of multiple epidemic waves using exogenous inputs

**Fig. 6:**
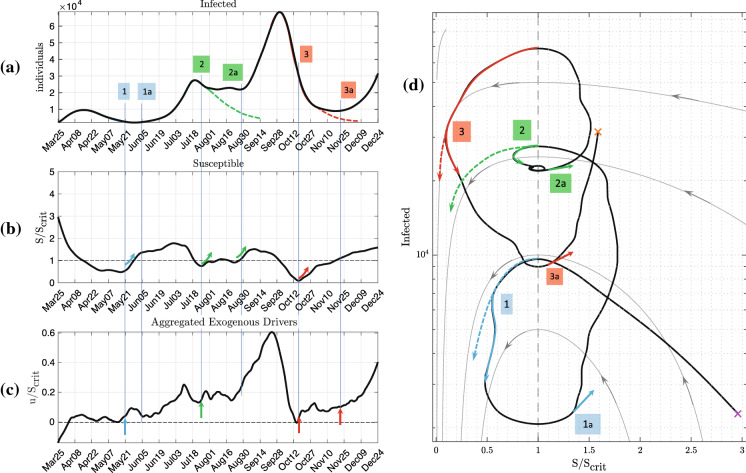
Analysis of the epidemic in the case of Israel. **a** Numbers of infected (measured and smoothed). **b** Numbers of susceptible normalized by $S_\text {crit}$. **c** Aggregated exogenous input normalized by $S_\text {crit}$. **d** Corresponding state portrait. Homogeneous solutions $u(t)=0$ shown as grey lines

The first example is Israel, which has experienced multiple waves of COVID-19 infections in 2020. Figure 6 shows the course of reported active infections in the period from March to December 2020. Each epidemic wave is highlighted in a different colour, including the extrapolated subsiding phases according to the conventional SIR model (dashed lines). Up to time point “1”, the first wave strictly follows the course as predicted by the basic SIR model.

Then, despite a still declining number of infections, from time point “1” onwards, the exogenous input *u*(*t*) steadily increases from 0 to values >0 which is followed by a rise in $S/S_{\text {crit}}$ and, eventually, by a rise in the number of infections about 2 weeks later (“1a”). Similarly, despite decreasing numbers of infections at time points “2” and “3”, *u*(*t*) is already on the rise, followed by the second and third epidemic waves with a delay of approximately 2 to 4 weeks (“2a” and “3a”). Hence, the predictive statement of the changes in *u*(*t*) can be used for the anticipation of the second and third waves about 2–4 weeks before their actual onsets.

This suggests that compartmental models in conjunction with exogenous inputs *u*(*t*) as a marker can be used for short-term predictions of epidemic waves. It should be noted again that for the estimation of the exogenous input *u*(*t*), the proposed method uses only the reported number of infections. Furthermore, the model parameters are estimated using data from the first surge only and are kept fixed for the remainder of the analysis. Hence, a CSIR model in combination with the exogenous input can be used as a measurement instrument, equivalent to a short-term “epidemometer”, which visualizes driving mechanisms underlying the epidemic development.

### Analysis of different countries

**Fig. 7:**
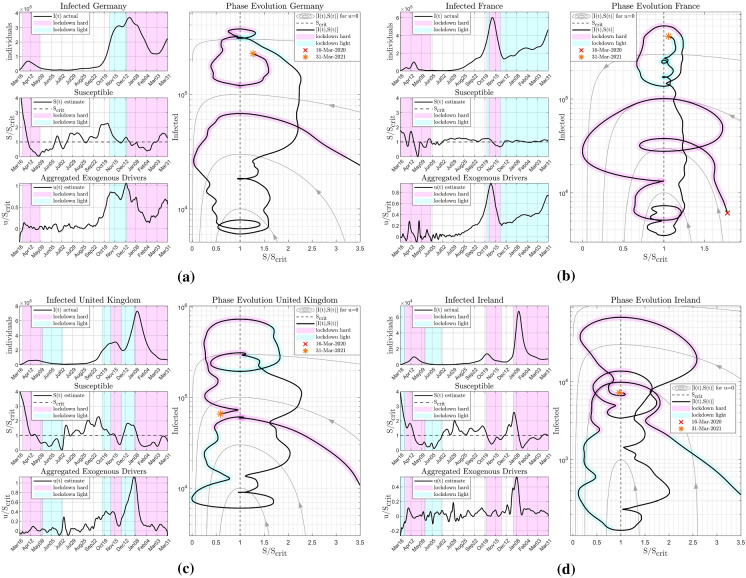
Pandemic courses in selected countries associated with non-pharmaceutical interventions. Germany (**a**); France (**b**); UK (**c**) and Ireland (**d**): Soft lockdowns (coloured in blue) and hard lockdowns (coloured in pink) affect infection numbers *I*, however, only with some time delay after susceptibility $S/S_{\text {crit}}$. Using the ratio of $u/S_{\text {crit}}$ as well as the state trajectories, critical developments of the epidemic can be detected much earlier

Next, the effects of governmental measures such as soft or hard lockdowns on the exogenous input *u*(*t*) are examined, enabling a quantitative assessment of their effectiveness in real time.

In Fig. 7, a respective analysis of the course of the epidemic in the period from March 2020 to January 2021 for selected countries (Germany, France, UK and Ireland) is given. Apart from the measured and smoothed infection numbers, estimates of the susceptibility *S* and the aggregated exogenous input *u*(*t*) using the proposed methodology are given. Periods, where light or hard lockdowns were in effect, are highlighted in colour.

As it has already been shown in the example of Israel, waves of infections are preceded by a growing exogenous input *u*(*t*). All hard lockdowns imposed in the respective countries were able to ease the epidemic waves by reducing the exogenous input *u*(*t*). Conversely, soft lockdowns were largely ineffective and did not have a significant effect on *u*(*t*) or drive $S/S_{crit}$ below the critical level of one.

From June onwards, infection numbers were consistently low, and the estimated exogenous input consistently stayed close to zero. Yet, the susceptibility *S*(*t*) is already close to $S_{crit}$ and as a result the infections start to increase again in August.

Clearly, one can see the role of the exogenous drivers *u*(*t*) and also of the susceptibility *S*: They can serve as early indicators for upcoming surges of infections and show immediate responses to governmental interventions. In addition to the three-time series, the state trajectory reveals even more clearly the current phase of the epidemic and whether another epidemic wave is to be expected.

Another advantage is that unexpected developments can be detected early on: In the graphs of the UK and Ireland, a steep increase in *u*(*t*) can be observed starting at the end of November and December 2020, respectively, reaching much higher values than seen in the past. This coincides with the first detections of a more contagious mutant VOC-202012/01 in the UK [51]. Note that again the rise of *u*(*t*) considerably precedes that of *I*(*t*).

### Correlation of $$S/S_crit $$ and $$R_eff $$

**Fig. 8:**
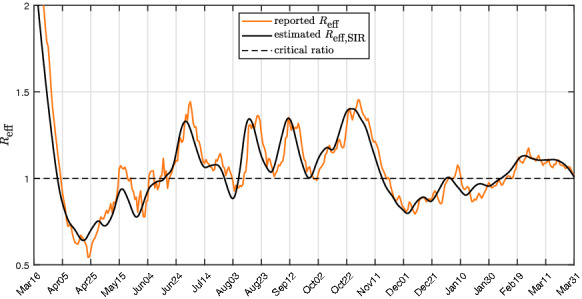
Comparison of $$R_eff $$ reported with $$R_eff,SIR $$ estimated based on $$S/S_crit $$ for Austria. A high correlation between the two approaches is visible

The effective reproduction number $$R_eff $$ can be utilized as a measure to describe the infection activity [52–54]. In the following, the similarities between the normalized number of susceptibles $$S/S_crit $$ and the effective reproduction number $$R_eff $$ are addressed, which is often based on statistical modelling [55]. Yet, both numbers indicate epidemic activity, which allows for a comparison. The reproduction number based on the SIR model can be obtained by

19 $$\begin{aligned} R_SIR (t) = \frac{S(t)}{S_crit }, \end{aligned}$$

with $$S_crit $$ of (5). The reported effective reproduction number $$R_eff $$ for Austria is compared in Fig. 8 to the estimated effective reproduction number for the SIR model $$R_eff,SIR $$ [56] based on $$S/S_crit $$.

$$R_eff $$ is calculated with statistical methods [57] and based on the reported governmental data [49]. The two signals show a strong observable correlation, but since they are derived from totally different approaches, they have to be interpreted accordingly. An advantage of the reproduction number based on $$S/S_crit $$ is, however, that it can be obtained in real time based on Eq. (10).

## Short-term forecast and analysis of intervention strategies

### Forecasting short-term epidemiological dynamics

**Fig. 9:**
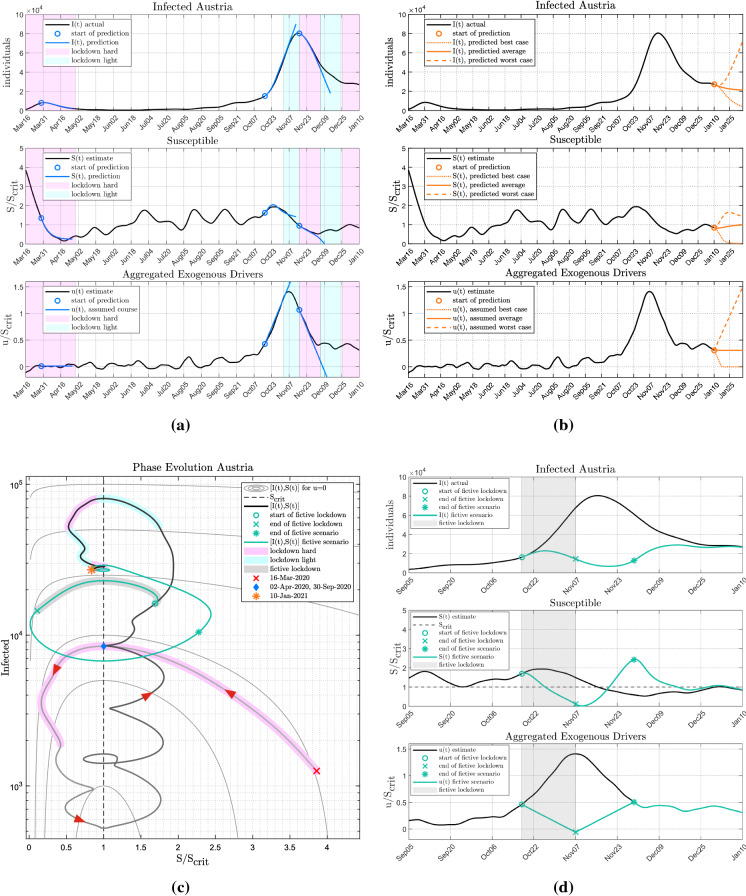
Analysis of short-term forecasts and intervention strategies. (**a**) Epidemic analysis of Austria along with three exemplary 4-week short-term predictions. For each prediction, *u*(*t*) is linearly extrapolated, as shown in the third subfigure. (**b**) Predicted future course of *S*(*t*) and *I*(*t*) for three different scenarios starting on January 4. The assumed trajectories for *u*(*t*) represent the best, the worst and an average case. (**c**) State diagram for Austria including a fictive scenario with earlier lockdown, green line). (**d**) A fictive scenario of a soft lockdown shifted forward to October 17 by applying the fictitious *u*(*t*) shown in the third subfigure

Figure 9a illustrates the feasibility of predictions in Austria. Three critical time points during the course of the epidemic are chosen: Shortly before the peak of the first wave at the end of March 2020, at the onset of the second wave and at the peak of the second wave, respectively. At each of these times, a 4-week prediction was made. The prediction is based on an assumption on the (unknown) future course of the exogenous drivers *u*(*t*). A linear projection on *u* is made, i.e. a linear continuation of the currently estimated trend, as seen in the third panel in Fig. 9a.

Figure 9a shows the resulting predictions of the susceptible *S* and of infection numbers *I* (blue line), respectively, plotted against the actually reported cases (black line). The first prediction at the end of March shows excellent agreement between the prediction and the actual numbers. The second prediction, starting in mid-October is also in a good agreement for the first 2 weeks of the prediction phase. After that, a light lockdown is imposed, stopping the linear growth of *u*(*t*) and leading to a decline in the infection cases. Similarly, for the third prediction phase, a good agreement can be observed for the first 2.5 weeks. After that, the trend of *u*(*t*) is again changed, this time by a lift of the hard lockdown.

Similarly, this approach is utilized to evaluate the impact of future measures starting at the 10th of January. Again, the presumed trajectories in Fig. 9b correspond to the effect of such measures that have been observed in the past. The bottom plot in Fig. 9b shows the assumed trajectory of *u*(*t*) for three different scenarios. The best case considers if *u*(*t*) decreases to zero. The average scenario shows that a constant *u*(*t*) also leads to constant *I*(*t*) in the near future. The worst case situation would be to assume a steep increase in *u*(*t*) again.

### Timing of non-pharmaceutical interventions (NPI)

The course of *u*(*t*) could also be used to predict and study fictitious courses of the epidemic triggered by different lockdowns.

The first question to be answered is *when* lockdowns should be considered within the course of an epidemic. In the centre of Fig. 9c, the blue diamond marks a state with the same number of infected individuals but occurring at two different points in time, April 2 and September 30, respectively. Starting exactly from the same initial state, the diverging trajectories of the epidemic in the phase plane following these 2 days are solely driven by *u*(*t*). While in spring *u*(*t*) was close to zero, on September 30 the beginning increase in *u*(*t*) triggered a strong increase in infections.

In view of the continued growth of both *u*(*t*) and $$S/S_crit $$ in the subsequent days, one could ask when a lockdown should have been imposed to prevent another epidemic surge. This is exemplified in Fig. 9d: The actual soft lockdown imposed in Austria on November 2 is fictitiously shifted forward in time to October 17. A prediction of infection numbers is obtained by assigning an *u*(*t*) associated with this particular intervention. Assuming that the impact of the lockdown would have been the same in terms of the exogenous drivers (cf. panel three in Fig. 9d), the prospective number of infections can be determined (cf. panel one in Fig. 9d). According to this model-based analysis, the number of infected individuals could have been reduced drastically and the second peak largely avoided. In this fictive scenario, the numbers $$S/S_crit $$ increase after the end of the lockdown due to the increasing *u*(*t*) (a complete reopening is assumed here) until the actual and fictive exogenous inputs are equal, which marks the end of the fictive lockdown. Therefore, $$S/S_crit $$ and consequently also *I*(*t*) from the fictive scenario eventually converge to the observed trajectories (after December 19).

## Conclusion

A methodology is presented in this work to analytically estimate an exogenous input *u*(*t*) to an epidemiological compartmental model, thus constructing an aggregated estimate of all exogenous drivers of an ongoing pandemic, resulting in a simple yet flexible model. The model captures linear infection surges [24] and recurrent pandemic surges based on the variations in the estimated input *u*(*t*). No additional knowledge of the actual or estimated effect of external drivers is needed and the aggregated effect of exogenous influences on the course of the pandemic is revealed in *u*(*t*). A suitable representation in the phase plane can be used for early detection of potentially critical developments that are otherwise not visible. The results indicate that in most cases the onset of a surge could be predicted around 2 weeks prior to the actual increase.

The estimation of the course of *u*(*t*) over time provides a real-time indication of the effect of current developments. The preceding course of *u*(*t*) together with absolute values of *I*(*t*) and *S*(*t*) is equalling an “epidemometer”, i.e. an early indicator of critical developments.

The predictive power of the proposed model can also be utilized to perform scenario simulations based on empirical evidence: The values and rates of the predicted *u*(*t*) can be based on earlier values for *u*(*t*) thus providing realistic assumptions for the simulations. Effects such as fading compliance of the population with NPIs can thus be included in a simulation.

The model parameters are obtained from data of infected individuals only, which allows for a straightforward and real-time monitoring of the pandemic situation. The basic model parameters are derived from the early phase of the epidemic. These model parameters remain fixed and require no further re-adjustments during the evolution of the epidemic. Unexpected and/or sudden critical developments such as the emergence of viral mutations become rapidly visible in *u*(*t*). Furthermore, it is shown that a time-varying infection rate $\beta (t)$ has much less explanatory power than the proposed approach.

In addition, the effective reproduction number $R_{\text {eff}}$ is provided and can be estimated by the proposed approach in real time. This is yet another common indicator of the pandemic situation [55], however, it can be delivered without any inherent delay.

With the currently available limited data the exogenous inputs *u*(*t*) cannot be dis-aggregated into specific components; an absolute measure of the effectiveness of non-pharmaceutical interventions is therefore not available. The simplicity of the CSIR model also constitutes a limitation: Possibly existing more complex pandemic dynamics will affect *u*(*t*), thus masking the effect of actual exogenous drivers. We are further aware of the fact that the proposed methodology might be extended and profit by comprising spatial and/or age-resolution. The methodology can therefore be applied to models with multiple compartments of infected and susceptibles as well [58].

In conclusion, this work demonstrates that flatness-based identification of unknown inputs in conjunction with established compartmental epidemiological models can elucidate the course of a pandemic with complex socio-economic interaction. As the proposed method is a generic approach, it can be applied to different epidemiological models as well as epidemic diseases other than COVID-19.

## Data Availability

The data used in this paper are based on the publicly available COVID-19 database of the Johns Hopkins University. In the case of Austria, the data are based on the Austrian Agency for Health and Food Safety (AGES).

## Appendix

### Consideration of the detection rate

An open question for the prediction of the COVID-19 epidemic is how the detected number of infected $I_{\mathrm{d}}(t)$ differ from the actual number of infected *I*(*t*). The time-dependent detection rate $\varphi (t)$ is related to the unknown, true number of infected *I*(*t*) as

20 $$\begin{aligned} I_\text {d}(t) = \varphi (t) I(t). \end{aligned}$$

A time-dependent detection rate $\varphi (t)$ can be included into model (2) and a suitable flat input *u*(*t*) can be derived, assuming a constant transmission rate $\beta$. The differentially flat system of Eqs. (6–7) is thus expressed by:

21 $$\begin{aligned} {\mathbf {x}}(t)&= {\mathbf {\Psi }}_{\mathbf {x}} \left( I_\text {d}(t),{\dot{I}}_\text {d}(t),{\ddot{I}}_\text {d}(t), \varphi (t), {\dot{\varphi }}(t),{\ddot{\varphi }}(t) \right) \end{aligned}$$

22 $$\begin{aligned} u(t)&= {\mathbf {\Psi }}_u \left( I_\text {d}(t),{\dot{I}}_\text {d}(t),{\ddot{I}}_\text {d}(t),\varphi (t), {\dot{\varphi }}(t),{\ddot{\varphi }}(t) \right) \end{aligned}$$

with some nonlinear functions ${\mathbf {\Psi }}_{\mathbf {x}}$ and ${\mathbf {\Psi }}_u$. The transmission rate and its derivatives are measured outputs of the system as well, as indicated by Eq. (8). Therefore, they are required to solve the flat system. Assuming that $\varphi (t)$ and its time derivatives are known, the first derivative of $I_\text {d}$ is

23 $$\begin{aligned} {\dot{I}}_\text {d} = {\dot{\varphi }} I + {\dot{I}} \varphi . \end{aligned}$$

Replacing the true numbers of infected $I,{\dot{I}}$ in the above equation yields the expressions for ${\dot{I}}_\text {d}$ as

24 $$\begin{aligned} {\dot{I}}_\text {d}&= \left( \frac{{\dot{\varphi }}}{\varphi } + \frac{S\beta }{N} -\gamma \right) I_\text {d}. \end{aligned}$$

Solving this equation for *S* yields the susceptibles as

25 $$\begin{aligned} S = \frac{N}{\beta } \left( \frac{{\dot{I}}_d}{I_d} - \frac{{\dot{\varphi }}}{\varphi } + \gamma \right) . \end{aligned}$$

The analytic expression for *u*(*t*) is obtained from the time derivative of ${\dot{I}}_\text {d}$ of Eq. (24) given by

26 $$\begin{aligned} {\ddot{I}}_\text {d} = \left( \frac{{\dot{\varphi }}}{\varphi } + \frac{\beta S}{N} - \gamma \right) {\dot{I}}_\text {d} + \left( \frac{{\ddot{\varphi }}}{\varphi } - \frac{{\dot{\varphi }}^2}{\varphi ^2} + \frac{{\dot{S}} \beta }{N} \right) I_\text {d}. \end{aligned}$$

By replacing ${\dot{S}}$ with ${\dot{S}}$ of model (2) and after rearranging the equation, the expression for the exogenous input is obtained as

27 $$\begin{aligned} u= & {} \frac{N}{\beta } \left( \frac{{\ddot{I}}_\text {d}}{I_\text {d}} - \left( \frac{{\dot{\varphi }}}{\varphi } + \frac{\beta S}{N} - \gamma \right) \frac{{\dot{I}}_\text {d}}{I_\text {d}} - \frac{{\ddot{\varphi }}}{\varphi } \right. \nonumber \\&\left. + \frac{{\dot{\varphi }}^2}{\varphi ^2} + \frac{\beta ^2 I_\text {d} S}{N^2 \varphi } \right) . \end{aligned}$$

Equation (27) collapses into Eq. (14) for $\varphi (t)$=1, ${\dot{\varphi }}$=${\ddot{\varphi }}$=0 and after replacing *S*. The source of $\varphi (t)$ is not a topic of this paper, but publications propose either a constant rate of detection [39], probabilistic distributions [59] or functions based on the positivity rates of tests.
